# Genetic diversity among *Plasmodium falciparum *field isolates in Pakistan measured with PCR genotyping of the merozoite surface protein 1 and 2

**DOI:** 10.1186/1475-2875-9-1

**Published:** 2010-01-01

**Authors:** Najia K Ghanchi, Andreas Mårtensson, Johan Ursing, Sana Jafri, Sándor Bereczky, Rabia Hussain, Mohammad A Beg

**Affiliations:** 1 Department of Pathology and Microbiology, Aga Khan University, Stadium Road, PO Box 3500, Karachi 74800, Pakistan; 2 Department of Biotechnology, University of Karachi, University Road, Karachi, Pakistan; 3 Malaria Research Lab, Infectious Diseases Unit, Department of Medicine, Karolinska University Hospital/Karolinska Institutet, Retziusväg 10, S-171 77 Stockholm, Sweden; 4 Division of Global Health (IHCAR), Department of Public Health Sciences, Karolinska Institutet, Nobelsväg 9, S-171 77 Stockholm, Sweden; 5 Centre for Microbiological Preparedness, Swedish Institute for Infectious Disease Control, Nobelsväg 18, SE-171 82 Stockholm, Sweden

## Abstract

**Background:**

The genetic diversity of *Plasmodium falciparum *has been extensively studied in various parts of the world. However, limited data are available from Pakistan. This study aimed to establish molecular characterization of *P. falciparum *field isolates in Pakistan measured with two highly polymorphic genetic markers, i.e. the *merozoite surface protein 1 *(*msp-1*)*and 2 *(*msp-2*).

**Methods:**

Between October 2005 and October 2007, 244 blood samples from patients with symptomatic blood-slide confirmed *P. falciparum *mono-infections attending the Aga Khan University Hospital, Karachi, or its collection units located in Sindh and Baluchistan provinces, Pakistan were collected. The genetic diversity of *P. falciparum *was analysed by length polymorphism following gel electrophoresis of DNA products from nested polymerase chain reactions (PCR) targeting block 2 of *msp-1 *and block 3 of *msp-2*, including their respective allelic families KI, MAD 20, RO33, and FC27, 3D7/IC.

**Results:**

A total of 238/244 (98%) patients had a positive PCR outcome in at least one genetic marker; the remaining six were excluded from analysis. A majority of patients had monoclonal infections. Only 56/231 (24%) and 51/236 (22%) carried multiple *P. falciparum *genotypes in *msp-1 *and *msp-2*, respectively. The estimated total number of genotypes was 25 *msp-1 *(12 KI; 8 MAD20; 5 RO33) and 33 *msp-2 *(14 FC27; 19 3D7/IC).

**Conclusions:**

This is the first report on molecular characterization of *P. falciparum *field isolates in Pakistan with regards to multiplicity of infection. The genetic diversity and allelic distribution found in this study is similar to previous reports from India and Southeast Asian countries with low malaria endemicity.

## Background

*Plasmodium falciparum *is an important public health problem in Pakistan causing at least 500,000 cases of malaria annually [1]. An increasing number of cases are being reported every year and the relative frequency of *P. falciparum *amongst slide positive malaria cases has increased from 45% in 1995 to 68% in 2006 [2-4]. In line with this, the National Malaria Control Programme has reported a six-fold increase in *P. falciparum *during the last decade. Despite this malaria has received scarce scientific attention, particularity with regards to molecular characterization of the local parasite population.

The introduction of PCR genotyping in malaria research has paved the way for major improvements in the understanding of parasite biology. For example, a number of highly diverse genetic markers of *P. falciparum *have been identified and extensively studied as potential vaccine candidates [5,6]. The most commonly used markers for genotyping of *P. falciparum *are the surface antigens *merozoite surface protein *1 (*msp-1*), *merozoite surface protein 2 (msp-2) *and the *glutamate-rich protein *(*glurp*) [7,8]. These three genetic markers are unlinked, i.e. located on different chromosomes, single copy genes with extensive polymorphism, both with regards to sequence and size, which is mostly generated by intragenic repeats that are variable in copy number and length of the repeat unit [9]. These features make them attractive candidates for studies where identification and enumeration of genetically distinct *P. falciparum *parasite sub-populations are of interest. As such they have proven to be useful tools both in molecular epidemiology studies in different epidemiological settings as well as to distinguish treatment failures from new infections in anti-malarial drug trials [10,11].

The genetic diversity among the *P. falciparum *population is an important indicator of the malaria transmission intensity in an area [12,13]. A high endemic area is generally characterized by extensive parasite diversity and infected humans often carry multiple genotypes. Conversely, the parasite population in a low transmission area has a limited genetic diversity and the majority of infections are monoclonal [14-17].

*Plasmodium falciparum *field isolates have been characterized in both Iran and India, but not previously in Pakistan, using the above mentioned molecular markers. This study, therefore, aimed to bridge this gap by molecular characterization of *P. falciparum *field isolates in Pakistan measured using two highly polymorphic markers, i.e. *msp-1 *and *msp-2*.

## Methods

### Study setting, participants, and ethics

Between October 2005 and October 2007, patients of all ages presenting with microscopy confirmed asexual *P. falciparum *mono-infections, regardless of parasite density and disease severity, at the Aga Khan University Hospital, Karachi or its established chain of health centres/collection units located in Sindh and Baluchistan provinces, Pakistan were enrolled in this study.

The study was conducted in accordance with the latest version of the Declaration of Helsinki and Good Clinical Practice [18]. Informed consent was obtained from all participants or in case of children from their parents/legal guardians. The study was approved by the ethical review committee of Aga University Hospital, Karachi, Pakistan.

### Blood collection and microscopy

An intravenous blood sample of 2 ml was collected in an EDTA tube in accordance with routine clinical practice for all patients referred for laboratory investigation of malaria infection. Initially, a blood smear was microscopically analysed using Leishman staining for screening of presence of malaria parasites [19]. In case of a positive screening slide a confirmatory thick and thin Giemsa-stained blood film was analysed for species identification and parasite density. For patients with confirmed *P. falciparum *mono-infection asexual parasites were counted against 200 white blood cells on the thick film. Parasite density was quantified (parasites/μl) by assuming an average of 8,000 leucocytes per μl blood [20]. All blood slides were examined by experienced microscopists at the clinical laboratory, Aga Khan University. Quality control was done for 10% of the slides by an independent microscopist blinded to the initial report.

For all patients enrolled in the study the remaining blood volume collected in the EDTA tube was transferred to cryovials and kept frozen at -80°C until used for DNA extraction. A brief epidemiological/demographic history was also collected from each participant using a structured questionnaire.

### DNA extraction and molecular analyses

Genomic DNA was extracted from a total of 200 μl whole blood per patient using the Qiagen DNA extraction kit (QIAGEN, USA) according to manufacturer's instructions. Nested PCR genotyping was performed both for the variable block 2 regions of *msp-1 *and block 3 of *msp-2*, considered to be the two most informative genetic markers for assessment of multiplicity of *P. falciparum *infection [9].

The initial amplification was followed by individual nested PCR reactions using family specific primers for *msp-1 *(KI, MAD20 and R033), and *msp-2 *(FC27 and 3D7/IC), respectively, based on previously described standard protocols [21]. Positive and negative controls were systematically incorporated in each PCR run. The *msp-1 *and *msp-2 *PCR products were loaded on 3% and 2% agarose gels, respectively, stained with ethidium-bromide, separated by electrophoresis and visualized under UV trans-illumination (GelDoc^®^, Biorad, Hercules, USA).

Analyses of number of genotypes and size polymorphism were digitalized using Quantity One^® ^software (Biorad, Hercules, USA). For assessment of the overall number of genotypes present within the *P. falciparum *population and their respective prevalence, individual genotypes were defined arbitrarily by binning 20 base-pair (bp) intervals together. The median genotype for each family of the respective genetic markers was identified. The absolute size of the identified median band +/- 10 bp formed the initial bin. Thereafter, each 20 bp interval below and above the median band were defined as representing a distinct genotype.

### Study design and statistical analyses

This was a descriptive/exploratory study, which precludes a power calculation of sample size. Data were entered in Microsoft Excel and exported to SPSS 15.0 software for analyses. Arithmetical means and medians, where applicable, were calculated for all continuous baseline demographic variables, except for asexual parasite density (geometric mean). Proportions of binary outcomes were compared using non-parametric tests. Spearman's rank correlation coefficients were calculated to assess association between multiplicity of infection and parasite densities. Statistical significance was defined as a *P *value ≤ 0.05.

## Results

### Baseline demographic data

A total of 244 patients with microscopy confirmed *P. falciparum *mono-infection were enrolled. Six (2.5%) were excluded from the analysis due to negative PCR outcome in both the *msp-1 *and *msp-2 *marker. The remaining 238 patients all presented with symptomatic malaria infection, 164 (69%) with uncomplicated and the remaining 74 (31%) with complicated/severe disease.

The geographical distribution of the 238 patients included in the analysis was: 176 (74%) from Karachi, 21 (9%) from Sukkur, 31 (13%) from rural areas of Sindh province and the remaining 10 (4%) from Baluchistan. The asexual *P. falciparum *parasitaemia ranged from 50 to 500,000 parasites/μl with a geometric mean of 8,412 parasites/μl.

### *Plasmodium falciparum *allelic diversity

Length polymorphism was assessed in 238 *P. falciparum *field isolates within the allelic families of *msp-1 *and *msp-2*, with a total of 289 distinct fragments detected, representing an estimated 25 *msp-1 *and 33 *msp-2 *genotypes. The distribution of genotypes within the respective allelic families of *msp-1 *and *msp-2*, as well as their corresponding bp ranges is presented in Table 1. All family specific allelic types of *msp-1*, i.e. MAD20, RO33 and KI, were observed in the different geographical locations. The proportions of MAD20, K1 and RO33 types were 46%, 26% and 14%, respectively. Some 14% of the infections carried two allelic types (MAD20/K1, RO33/K1, MAD20/RO33), whereas no sample contained all three allelic types of *msp-1*. The occurrence of K1 family specific alleles of *msp-1 *ranged from 30-65% with the highest proportion in Baluchistan, whereas MAD20 allelic family was detected in 20-61% of the samples. The RO33 allelic type of *msp-1*, which was observed in 20% of the samples, was comparatively less prevalent as well as less polymorphic. For *msp-2 *allelic families 44% of the infections carried FC27 type and 40% carried 3D7/IC type, whereas 16% of the infections harboured both allelic types. The two allelic types of *msp-2 *were equally distributed with proportion of 3D7/IC and FC27 ranging between 46-70% and 30-69%, respectively, in isolates from different geographic locations.

**Table 1:** Number of *Plasmodium falciparum *genotypes and base pair ranges observed in *msp-1 *and *msp-2*, as well as their respective allelic families

	*msp-1*			*msp-2*	
	
	K1	MAD20	RO33	FC27	IC
Base pair range	145-459	100-257	108-196	205-512	310-894
No. of genotypes	12	8	5	14	19
Total no. of genotypes	25			33	
Overall multiplicity of infection	1.25			1.22	

### Association between mean multiplicity of infection, parasite densities and age

The overall mean multiplicity of *msp-1 *and *msp-2 *genotypes per infection were 1.25 (95% CI: 1.19-1.31) and 1.22 (95% CI: 1.17-1.28), respectively. The multiplicity of infection observed in the three different geographical areas is presented in Table 2. There was no statistical difference in multiplicity of infection in patients originating from Karachi and Sindh (p = 0.670). However, the low number of patients from Baluchistan (n = 10) precludes a meaningful comparison of the diversity in this region with the other two.

**Table 2:** Multiplicity of *Plasmodium falciparum *infection as assessed with the *msp-1 *and *msp-2 *marker in different geographic locations of Southern Pakistan

Number of PCR fragments	Karachi (%)	Sindh (%)	Baluchistan (%)
*msp-1*			
1	127 (74)	40 (77)	8 (89)
2	41 (24)	12 (23)	1 (11)
3	2 (1)	0	0
Multiplicity of infection*	1.26	1.23	1.11
*msp2*			
1	135(77)	40 (77)	10 (100)
2	38 (22)	11 (21)	0
3	1 (0.5)	1 (2)	0
Multiplicity of infection*	1.23	1.25	1.00

* Mean multiplicity of infection

A majority of the infections were monoclonal. Only 56/231 (24%) and 51/236 (22%) patients carried multiple *P. falciparum *genotypes in *msp-1 *and *msp-2*, respectively. *P. falciparum *parasite density stratified by number of *msp-1 *and *msp-2 *genotypes per infection is presented in Table 3. The asexual parasite density (geometric mean) was significantly higher (21,210 parasites/μl) in the 56 patients carrying multiple *msp-1 *genotypes compared with the 175 patients with monoclonal infections (7,049 parasites/μl, p < 0.001). However, no statistically significant difference was observed between the 51 patients harbouring multiclonal and the 185 patients with single *msp-2 *genotype infections, 10,532 versus 7,793 parasites/μl, respectively (p = 0.519). A statistically significant correlation between parasite density and number of genotypes was observed in *msp-1 *(Spearman rank coefficient = 0.184; p = 0.005), but not in *msp-2 *(p = 0.432). Parasite density and mean multiplicity of infection in *msp-1 *and *msp-2 *stratified by age is presented in Table 4, with no statistically significant differences observed.

**Table 3:** *Plasmodium falciparum *parasite density stratified by number of *msp-1 *and *msp-2 *genotypes per infection.

Number of PCR fragments*		*msp-1*		*msp-2*
	
	N^§^	Parasite density (parasites/μl)	N	Parasite density (parasites/μl)
1	166	7049	170	7793
2	48	20160	44	9721
3	2	71746	2	61399

* Only samples with positive PCR outcomes in both *msp-1 *and *msp-2 *were included.

^§ ^Parasite densities were available form 216 patients.

**Table 4:** *Plasmodium falciparum *parasite density and mean multiplicity of infection in *msp-1 *and *msp-2 *stratified by age

Age (years)	N*	Parasite density (parasites/μl)	*msp-1*	*msp-2*
< 3	12	3620	1.25	1.17
3-5	16	6116	1.31	1.25
6-10	23	5760	1.26	1.35
11-15	15	5490	1.13	1.07
16-30	71	10281	1.20	1.30
> 30	79	10312	1.19	1.16

*Parasite densities were available form 216 patients.

## Discussion

This is the first report on genetic diversity of *P. falciparum *field isolates from Pakistan and as such, it bridges an important gap in the understanding of the molecular characteristics of the parasite population in South Asia.

The results reveal a relatively limited genetic diversity and multiplicity of infection. These findings are compatible with the general belief that Pakistan, despite potential substantial regional variations and recent increases in *P. falciparum *incidence, represents a low malaria transmission setting [13,14,16,22,23]. Moreover, the results are in line with several previous reports from India, Iran and Southeast Asian countries with low malaria transmission [24]. In contrast, the results of this study do not corroborate recent finding from Iran where Zakeri *et al *reported a relatively high multiplicity of infection in both the *msp-1 *and *msp-2 *marker from a presumably low endemic area [21].

However, it should be emphasized that a majority of patients (74%) presented with symptomatic malaria infection at a tertiary level of care, i.e. the Aga Khan University Hospital. Consequently, some of these patients may have received anti-malarial treatment prior to enrolment in the study. As treatment is likely to reduce the number of genotypes in an infected individual, the *P. falciparum *identified in these patients may not be representative for the Pakistani parasite population. Moreover, reports of genetic diversity from low endemic areas generally study symptomatic *P. falciparum *infections, since asymptomatic carriers rarely are observed in setting where semi-immunity cannot be acquired [25]. This is of importance since data indicate that symptomatic infections generally appear to harbour a lower multiplicity of infection as compared to asymptomatic children residing in high transmission areas in Africa [26]. Lastly, the inability to detect all parasite sub-populations present in an individual with one single blood sample, either due to inborn limitations of the PCR technique to detect minority clones or parasite population dynamics, may also be considered as a factor potentially underestimating the multiplicity of infection retrieved in our study [27]. Thus, the results of this study may reflect a significant underestimation of the genetic diversity of the *P. falciparum *population in Pakistan.

The reported study represents a first attempt to establish molecular techniques for malaria research at the Aga Khan University, Karachi. Future studies will be designed to geographically expand the collection of *P. falciparum *field isolates to include all four provinces of Pakistan. During the next phase, analyses of genetic markers related to anti-malarial drug resistance will also be included to gain a more thorough picture of *P. falciparum *molecular epidemiology in Pakistan.

## Conclusions

The genetic diversity and allelic distribution found in this study is similar to previous reports from India and Southeast Asian countries with low malaria endemicity.

## Competing interests

The authors declare that they have no competing interests.

## Authors' contributions

NKG performed DNA extractions and PCR genotyping, data entry, analysis and interpretation and drafted the first version of the report. AM designed and planned the study, performed data analysis and interpretation and wrote the report. JU performed data analysis and interpretation and participated in writing of the report. SB and RH participated in data analysis and interpretation as well as with writing of the report. SJ performed PCR genotyping and data analyses. MAB designed and planned the study, performed data analysis and interpretation and wrote the report. All authors read and approved the final manuscript.
